# Meeting of Dental Society of Western New York

**Published:** 1863-07

**Authors:** Leon F. Harvey


### Proceedings of Societies.

### SEMI-ANNUAL MEETING OF THE DENTAL SOCIETY OF WESTERN NEW YORK.

REPORTED BY LEON F. HARVEY, M. D.

This Society held its first semi-annual meeting in Rochester on Tuesday, May 5th, 1863.

President, Dr. C. W. Harvey, in the Chair.

Minutes of the last meeting read and adopted.

On motion, Dr. Leon F. Harvey was invited to report the proceedings of the Society for publication.

Dr. S. G. Wood read an interesting essay.

On motion, a vote of thanks was given to Dr. Wood for his able paper, and a copy requested for publication.

The committee appointed at the last meeting presented the following subjects for discussion:

I. Obtaining Impressions.

II. Obtaining Articulations.

III. The Air-Chamber.

IV. The Relative Merits of Different Materials as a Base for Artificial Teeth.

V. Filling Teeth.

VI. Correcting Irregularities.

VII. Dental Etiquette.

Dr. Whitney always uses plaster for taking impressions for full sets; never relies on wax. In partial cases uses wax or plaster. When using plaster, generally draws it out while yet soft; for which idea he is indebted to Dr. Geo. B. Snow.

Dr. E. L. Wood always takes impressions with wax. After an experience of twenty years, prefers wax to plaster.

Dr. E. G. Ward uses plaster in whole cases, and generally in partial cases. Allows the plaster to become quite hard. Threw wax away long ago.

Dr. Geo. B. Snow prefers to draw the plaster when soft. The cup should fit the mouth accurately; the back part should be built up with some soft material. Fine plaster will set quicker, and not run as much as coarse. The patient's head should be thrown very far forward, so that the plaster will not touch the soft palate.

Dr. Whitney usually takes the impression in wax first, and then pares out enough to use two teaspoonfuls of plaster. Uses superfine plaster with warm water and a little salt.

Dr. Bristol would like to know if there is any use in the air-chamber? Is not prepared to condemn it. Has found good results with and without.

Dr. Gaskill thinks the air-chamber of great benefit. Uses the semi-circular shape.

Dr. Belding considers the air-chamber very beneficial. Had a case where the membrane was very soft and yielding. Tried twice without a chamber, but could not succeed. Then used a large air-chamber, and the patient found it difficult to get the plate out. Would not use it in a good mouth. Has used two air-chambers.

Dr. L. D. Walters has always used it. After four weeks it fills up, and is of no use. Has made them both shallow and deep.

Dr. Gaskill doubted that it fills up in four weeks.

Dr. Wilson has used the air-chamber, but is not in favor of it. Can always succeed without it with a good impression. It fills up in a fleshy mouth. In answer to Dr. Snow, he says that he removes a little plaster from the sides of the cast in a soft mouth.

Dr. E. L. Wood always prefers to make a plate without the air-chamber. Finds that the chamber fills up in four weeks.

Dr. Belding thinks that the chamber never fills entirely.

Dr. Rockway has abandoned the air-chamber. Now makes a slight vacuum. After taking the impression, rubs off with the finger the highest part in a soft mouth. In a hard mouth, fits it perfectly.

Dr. Whitney.—When the impression is poor, the air-chamber is useful; when it is good, the chamber is worthless.

Dr. C. W. Harvey has used plates with and without the chamber. Considers it of no use, except to allow the patient to become accustomed to the plate.

Dr. Crittenden once thought he could not succeed without the chamber, but soon found the mouth became spongy. Has not used it for three or four years.

Dr. Bristol considers rubber of all bases the best. Is sorry to say it, as it is a curse to the profession.

Dr. Whitney agrees with Dr. Bristol.

Dr. C. W. Harvey has found that decay does not take place where rubber clasps are adjusted to the teeth in partial sets. Thinks that rubber will supersede all other materials.

Dr. Bristol wished to know if rubber hardened after it was finished? Thinks it does. Has found that plates at first will spring, and after a time will break.

Dr. E. L. Wood says it constantly hardens. Would like to know if any patient has been known to have paralysis of the face from wearing a full case of rubber teeth?

Dr. Bristol had such a case; but thinks it was from pressure on a muscle very low down. Mechanical pressure only could produce it.

Dr. E. L. Wood has patients who have an unpleasant feeling and heat about the mouth. Would like to know if it is due to rubber?

Dr. Mix has a case in which the lady, who wears an upper and lower case of teeth, can wear the rubber but a short time without an unpleasant feeling and heat. She wears a partial case. Feels a galvanic action when the teeth strike.

Attributes the heat to the fact that the patient has had thrush in the mouth.

Dr. Whitney said that there is great difference in the kind of rubber; that some was manufactured better than others.

Dr. Bristol has had patients wearing rubber plates, who complained of heat in the mouth, and red patches have appeared on the lips and jaws. Upon the use of gold plate, the trouble disappeared.

Dr. E. F. Wilson.—A young lady patient of his wore an upper and lower gold set of teeth; her gums were as red as a beet. Put in a rubber upper set, and the gum healed, but the lower continued sore. Changed the lower for rubber, and that healed also. Finds that when the rubber is not corked enough, we have the soreness and redness. Thinks finished rubber will harden in the case, but not as fast in the mouth.

Dr. Geo. B. Snow has seen redness when rubber was worn.

Dr. L. D. Walter uses the rubber. Likes gold the best. Would be glad if rubber was out of the way. Thinks it hardens after being finished. Has seen rubber plates worn eight years, and are good now. Considers it durable.

Drs. Wilson and Blakesly had seen plates worn five to eight years, and they were as sound as when made.

Dr. L. F. Harvey wished to know the cause of blisters on a plate after mending?

Dr. Geo. B. Snow thinks it is due to the mucus on the plate; the steam presses the plaster away, and the rubber expands.

Dr. Blakesly wished that the Hard Rubber Co. could be induced to give us better rubber. Has seen bad results from it.

Dr. Squiers found the redness of mouth in a lady, who had been salivated, who wore a rubber plate.

Dr. Bristol said that continuing finished rubber plates in alcohol for a time bleached them.

Dr. Blakesly finds that if rubber is left in alcohol too long, it will draw away from the teeth. Has a tendency to soften.

Dr. E. L. Wood considers that rubber left in alcohol, even twenty-four hours, becomes denser and firmer.

Dr. Whitney thinks dentists fail from faulty articulations, even when they have good fitting plates. Is obliged to grind the grinding surface of teeth, which should not be done. Finds trouble in procuring the right bite.

Dr. Geo. B. Snow considers this the most difficult part of dentistry. Has found that patients with their heads on the rest can not bite naturally.

Dr. Blakesly tells his patients to shut the lower teeth behind the upper on the wax. Also tells them to swallow, and allow the mouth to remain closed. Succeeds well in this way.

Dr. Bristol is in the habit of making a set of teeth in wax, and allowing the patient to bite into it. Is obliged to grind the grinding surfaces a great deal.

Dr. Geo. B. Snow said that a wheel of Scotch stone would take off the roughness due to grinding.

Dr. E. L. Wood considers that dentists should never grind teeth when all are artificial. Uses Whitney's flask. Fine India vermilion is good to color bone filling for points between the teeth.

Dr. Squires asked for a little information in regard to temporary plates.

Dr. Whitney says that he asks as much for temporary as for permanent work. Wants patients to forget that there is such a thing as temporary work.

Dr. E. L. Wood makes his temporary work as poor as possible, so that patients will be obliged to get new plates very soon.

On motion, Drs. L. D. Walter, C. W. Harvey, L. W. Bristol, Barbour, and Geo. B. Snow were appointed a committee to demonstrate filling at the next meeting of the Society, the dentists residing at the place of meeting to be requested to furnish patients.

On motion, each member of the Society was requested to exhibit a specimen in dentistry at the next meeting of the Society.

Dr. Geo. B. Snow presented a case where he had moved the incisors outside of the lower teeth with a rubber plate, made to fit the roof of the mouth, the rubber running over the molars to bite upon. Wooden pegs were placed in holes drilled in the rubber opposite the incisors. Removed the pegs every few days.

On motion, a vote of thanks was given Dr. Snow for the simple and ingenious apparatus he presented.

Dr. Whitney thinks there is danger in commencing to regulate when children are quite young. May destroy the teeth before the apex is fairly formed. Uses the inclined plane.

Dr. Bristol considers that from eight to twelve years of age is the best time to move teeth. It is an operation that never pays for the trouble and anxiety attending it.

After a discussion upon the subject of "Dental Etiquette," the following resolution was passed:—

*Resolved*, That the members of this Association, in recommending patients to other dentists, always recommend the regular members of this or some other association.

On motion, a committee of three, consisting of Drs. Whitney, Walter, and L. F. Harvey, were appointed to prepare subjects for discussion at the October meeting.

On motion, a committee of two, consisting of Drs. L. F. Harvey and Geo. B. Snow, were appointed to prepare essays to be read at the next meeting.

On motion, the members were invited to read papers on the subjects which the committee prepare for discussion.

On motion, the Society adjourned to meet at Buffalo.

